# Early Detection and Age-Comparative Analysis of Colorectal Cancer Screening: Insights from the Turkish Population

**DOI:** 10.3390/curroncol32030153

**Published:** 2025-03-06

**Authors:** Cem Batuhan Ofluoğlu, Fırat Mülküt, İsa Caner Aydın, Mehmet Karahan

**Affiliations:** 1 Department of Gastroenterology Surgery, Sancaktepe Training and Research Hospital, University of Health Sciences, 34785 Istanbul, Turkey; 2 Department of General Surgery, Sancaktepe Training and Research Hospital, University of Health Sciences, 34785 Istanbul, Turkey; firatmulkut@hotmail.com; 3 Ministry of Health, Department of Gastroenterology Surgery, Zonguldak Ataturk State Hospital, 34147 Zonguldak, Turkey; isacaner.aydin@saglik.gov.tr; 4 Department of General Surgery, Kartal Dr. Lütfi Kırdar City Hospital, University of Health Sciences, 34130 Istanbul, Turkey; mehmet.karahan2@saglik.gov.tr

**Keywords:** colorectal cancer screening, early-onset colorectal cancer, diagnostic yield, advanced neoplasia, Turkish population

## Abstract

Background: This study aimed to evaluate the diagnostic yield of colonoscopy in asymptomatic individuals aged 45–49 years compared with those aged 50–54 years in a Turkish population, providing insights into region-specific screening strategies. Methods: This retrospective multicenter study was conducted across three tertiary endoscopy units in Turkey. Screening colonoscopy data from 3943 asymptomatic individuals aged 45–54 years between 2018 and 2023 were analyzed. The patients were stratified into two groups: 45–49 years (Group 1) and 50–54 years (Group 2). Demographic characteristics, polyp size, histological features, and prevalence of early-onset advanced colorectal neoplasia (EAO-aCRN) were assessed. Results: A total of 3943 patients were included, with 862 in Group 1 (45–49 years) and 3081 in Group 2 (50–54 years). The polyp detection rate was 16.6% in Group 1 and 22.9% in Group 2 (*p* < 0.001). The adenoma detection rates were 10.8% and 13.9% in Groups 1 and 2, respectively (*p* = 0.018). The advanced polyp detection rates were 3.2% and 7.3% in Groups 1 and 2, respectively (*p* < 0.001). Mean polyp size was 6.5 ± 5.1 mm in Group 1 and 8.8 ± 8.4 mm in Group 2 (*p* < 0.001). The mean number of polyps per patient was 1.5 ± 0.8 in Group 1 and 1.9 ± 1.6 in Group 2 (*p* = 0.023). Advanced neoplasia was detected in 16.6% of Group 1 patients compared with 22.9% of Group 2 patients (*p* < 0.001). Conclusions: While CRC screening at age 45 demonstrated lower detection rates of polyps and advanced neoplasia than at age 50, the higher prevalence of EAO-CRN among 45–49-year-olds in Turkey underscores the importance of early screening in high-risk populations. Tailored regional strategies incorporating individual risk factors are crucial for optimizing CRC prevention policies.

## 1. Introduction

Colorectal cancer (CRC) is a leading cause of cancer-related morbidity and mortality worldwide, with increasing incidence among individuals aged <50 years. This phenomenon, referred to as early-onset colorectal cancer (EOCRC), has sparked debate regarding the optimal age to initiate screening [1,2]. In response to these rising trends, organizations such as the U.S. Preventive Services Task Force (USPSTF), the American Cancer Society, the National Comprehensive Cancer Network (NCCN), and the American College of Gastroenterology recommend lowering the screening age to 45 [3,4], while the American Academy of Family Physicians (AAFP) still recommends starting at 50 [5,6,7]. Including patients aged 40–50 in screening could allow earlier intervention before invasive carcinoma develops, but some studies argue that this may increase costs without significantly influencing prognosis [8]. Despite these updates, evidence supporting the effectiveness of lowering the screening age in specific populations remains limited, particularly in regions with unique demographic and healthcare characteristics. Studies have consistently demonstrated the rising burden of CRC in younger cohorts, with a disproportionately high prevalence of distal and rectal cancers among individuals aged 45–49 years [9,10]. However, questions persist regarding the cost-effectiveness, feasibility, and diagnostic yield of widespread screening at this younger age. Early-onset colorectal neoplasia (EAO-CRN) refers to all neoplastic lesions detected in individuals aged <50 years [2]. In contrast, early-onset advanced colorectal neoplasia (EAO-aCRN) is defined as adenomas ≥ 10 mm, high-grade dysplasia, villous/tubulovillous histology, carcinoma in situ, or multiple adenomas (≥3). The prevalence of early-onset advanced colorectal neoplasia (EAO-aCRN) in younger populations has become a critical metric for assessing the burden of advanced lesions in individuals below the traditional screening age [3,4].

This study aimed to assess the diagnostic yield of colonoscopy among asymptomatic individuals aged 45–49 years compared to the currently recommended age group of 50–54 years in the Turkish population to provide region-specific evidence to inform screening policies and improve CRC prevention strategies. The secondary objectives were to evaluate the prevalence of EAO-CRN and EAO-aCRN, analyze the anatomical distribution of colorectal lesions, classify detected polyps based on histological features, and determine the potential impact of early screening on colorectal cancer prevention strategies.

## 2. Material and Methods

### 2.1. Ethical Approval and Study Design

This retrospective multicenter study was conducted at three tertiary endoscopy units (C1, C2, and C3) after receiving ethical approval from the ethics committee of the S.B.U. Sancaktepe Dr. Ilhan Varank Training and Research Hospital (Decision No: 2024/45; date: 14 February 2024). Data from colonoscopies performed between January 2018 and December 2023 were reviewed. Following the USPSTF recommendations in 2021 to lower the screening age for colorectal cancer (CRC) to 45 years, screening data from patients aged 45–49 years were incorporated into the study to assess diagnostic yield at a younger age threshold.

### 2.2. Study Population

Patients aged 45–54 years who underwent an asymptomatic screening colonoscopy were included in this study. They were stratified into two groups: Group 1 (45–49 years), patients screened according to the lower screening age, and Group 2 (50–54 years), patients screened under the previously established guidelines. The selection of the 45–49 and 50–54 age groups was based on global trends, indicating an increasing burden of EOCRC among individuals aged <50 years [5,6].

Exclusion criteria included suboptimal bowel preparation (classes 0–1 according to the Boston bowel preparation scale), history of adenomas, colorectal surgery or malignancy, diagnostic colonoscopies for symptomatic cases, and age < 45 or above 54 years. Patients with a family history of CRC were excluded to prevent selection bias and ensure results reflect the general screening population, avoiding an increased detection rate of advanced neoplasia and skewed results. This study did not include individuals aged 40–44 years, as routine CRC screening is not performed in asymptomatic individuals within this age range.

### 2.3. Data Collection

Demographic characteristics and histopathological findings were also extracted. The parameters analyzed included polyp presence, size, localization, and histology as well as advanced lesions defined as adenomas ≥ 10 mm, high-grade dysplasia, villous or tubulovillous histology, carcinoma in situ, or more than three adenomas. Due to the retrospective nature of this study, data on lifestyle-related risk factors (dietary habits, smoking status, and metabolic syndrome) were not evaluated.

### 2.4. Endoscopy Procedure

All colonoscopies were conducted by colorectal surgeons with a minimum of five years of experience in performing screening colonoscopies. Colonoscopy was performed using a Fujifilm EC-600WM colonoscope (Fujifilm, Tokyo, Japan) equipped with Blue Light Imaging (BLI) and Linked Color Imaging (LCI) technologies. These image-enhanced endoscopy (IEE) modalities were selectively employed to enhance polyp characterization and mucosal pattern differentiation.

### 2.5. Statistical Analysis

Statistical analyses were performed using SPSS version 26.0. Continuous variables are expressed as mean ± standard deviation, and categorical variables are expressed as percentages. The Kolmogorov–Smirnov test was performed to validate the assumptions of normality for continuous variables. The Mann–Whitney U test was conducted alongside the t-tests to confirm the robustness of the comparisons. The chi-squared test was used for categorical variables. Statistical significance was set at *p* < 0.05.

## 3. Results

A total of 32,848 colonoscopies were performed on patients aged ≥18 years during the study period. Of these, 24,251 were excluded because they were either <45 or >54 years of age. Among the remaining 8597 patients, 4324 were excluded because they underwent colonoscopy for diagnostic purposes related to specific symptoms. A total of 4273 patients remained in this study. Subsequently, 330 patients were excluded for the following reasons: missing data (n = 11), incomplete evaluations (n = 268), repeated colonoscopies (n = 8), family or personal history of colorectal cancer (n = 13), and evaluations conducted for polyp surveillance (n = 30).

**Figure 1:**
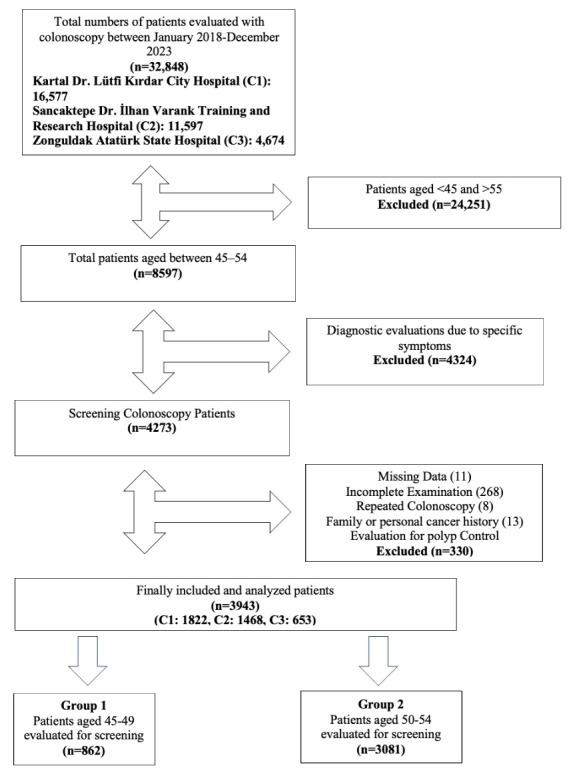
Flowchart of patient enrollment.

Ultimately, 3943 patients met the inclusion criteria and were divided into study groups. These patients were distributed across three participating centers: 1822 from C1, 1468 from C2, and 653 from C3. Group 1 consisted of 862 patients aged 45–49 years, whereas Group 2 included 3081 patients aged 50–54 years. A detailed patient enrollment flowchart is presented in Figure 1.

The sex distribution was comparable between Group 1 (45–49 years) and Group 2 (50–54 years), with males comprising 61.5% and 61.1% of the groups, respectively, and females comprising 38.5% and 38.9%, respectively. No statistically significant differences were observed between the groups (*p* = 0.839). Significant differences were noted in the distribution of polyp localization between the two groups (*p* < 0.001). In Group 1, the majority of polyps were localized to the left colon (47.5%). In contrast, Group 2 exhibited a higher prevalence of multiple localizations (31.3%) followed by the left colon (26.7%). The distribution of adenomatous histology differed significantly between the groups (*p* = 0.012). Tubular adenomas were more prevalent in Group 2 (88.4% vs. 84.0%), whereas tubulovillous adenomas were slightly more common in Group 1 (13.6% vs. 11.6%). Villous adenomas were exclusively identified in Group 1 (2.5%). No significant differences were observed in non-adenomatous histology (*p* = 0.094) or prevalence of serrated polyps (*p* = 0.645) between the groups. The polyps were significantly larger in Group 2 (8.8 ± 8.4 mm) than in Group 1 (6.5 ± 5.1 mm; *p* < 0.001). Group 2 had a significantly higher mean number of polyps per patient (1.9 ± 1.6) than Group 1 (1.5 ± 0.8; *p* = 0.023) (Table 1).

The detection rates for polyps, adenomas, and advanced polyps demonstrated statistically significant differences between Group 1 (45–49 years) and Group 2 (50–54 years). Group 2 exhibited a significantly higher polyp detection rate (22.9%) than Group 1 (16.6%) (*p* < 0.001). The adenoma detection rate was also significantly higher in Group 2 (13.9%) than in Group 1 (10.8%) (*p* = 0.018). Group 2 showed a significantly higher detection rate of advanced polyps (7.3%) than Group 1 (3.2%) (*p* < 0.001) (Table 2).

Univariate regression analysis was performed to analyze the polyp subtypes, size, and anatomical distribution. Left colon polyp development was significantly more frequent in the 45–49 age group (OR: 1.699, *p* = 0.020). Multiple-site polyp development was observed at a significantly lower rate in this age group (OR 0.702, *p* = 0.044). No significant differences were detected in the prevalence of small polyps (0–4 and 5–9 mm) between the study groups. However, polyps ≥ 10 mm were significantly less frequent among individuals aged 45–49 years (OR, 0.476; *p* < 0.001). Multivariate logistic regression analysis demonstrated that overall polyp development (odds ratio [OR]: 0.515, *p* < 0.001) and adenomatous polyp development (OR: 0.643, *p* < 0.001) were significantly lower in individuals aged 45–49 years than in the reference group. Regression analysis results for polyp development and histological subtypes among individuals aged 45–49 years are presented in Table 3.

## 4. Discussion

This study evaluated the necessity of lowering the screening age for CRC by analyzing the prevalence of EAO-CRN and EAO-aCRN in individuals aged 45–49 years (younger population) and 50–54 years (older population) in a Turkish cohort.

The prevalence of EAO-CRN in Group 1 (16.6%) exceeded the global pooled prevalence for individuals aged 45–49 years (13.7%, 95% CI: 11.2–16.8%), as reported by Li et al., Meng et al., and Ong et al. [7,8,9]. Similarly, the advanced neoplasia detection rate in the younger population (3.2%) was higher than the global average of 2.2% (95% CI: 1.6–3.1%), as estimated in the Global Burden of Disease 2021 Study (GBD 2021) [9,10]. In the older population, the EAO-CRN prevalence of 22.9% was consistent with global pooled data for individuals aged 50–59 years (24.8%, 95% CI: 19.5–30.8%), as reported in the GBD Study and BMC Public Health Analysis [7,9]. However, the advanced neoplasia detection rate in the older population (7.3%) exceeded the global benchmark of 4.2% (95% CI: 3.1–5.7%), in agreement with the findings of Ong et al. [8].

Furthermore, the CRC prevalence in our younger population (6.9%) was markedly higher than the global pooled prevalence in individuals aged 45–49 years (0.05%, 95% CI: 0.00029–0.0008%), as reported by Meng et al. [9]. In the older population, CRC prevalence (11.8%) was also significantly higher than the global data for individuals aged 50–59 years (4.2%, 95% CI: 3.1–5.7%) [5,8,9]. These discrepancies may reflect unique regional risk factors such as dietary patterns and genetic predispositions.

Supporters of lowering the screening age argue that CRC and metastatic disease are increasing, with 10–11% of CRC cases occurring before the age of 50 years. They suggest that colonoscopies performed more frequently in those under 50 years of age could detect more cases before disease progression. Meanwhile, those favoring the current screening age point out the higher CRC development risk in older populations (34 vs. 60:100,000) and argue that lowering the age ignores individual risk factors such as sex, diabetes, diet, and lifestyle, which are better addressed by statistical models than randomized trials [11,12,13]. Another study that evaluated patients aged 45–49 and 50–54 found that CRC incidence was significantly lower in those who underwent colonoscopy in both groups, supporting the recommendation to lower the screening age to 45 years. Data from patients in these age groups in Florida showed that 23.7% of 45–49-year-olds and 15.7% of 50–54-year-olds underwent colonoscopy, with these rates applied to both screening and those previously diagnosed with polyps [9]. The detection of significant neoplasia in individuals aged 45–49 aligns with recent recommendations by organizations, such as the American Cancer Society and the U.S. Preventive Services Task Force to initiate CRC screening at age 45 [14]. Although the prevalence of polyps, adenomas, and advanced polyps was lower in the younger population than in the older population, the higher prevalence of left-sided and rectal lesions in younger individuals (63.8% vs. 44.9%; *p* < 0.001) suggests that targeted screening strategies such as flexible sigmoidoscopy or left-sided colonoscopy may be beneficial in resource-constrained settings.

The prevalence of EAO-CRN in Turkey’s younger population (16.6%) was notably higher than that in East Asia (13.4%, 95% CI: 10.3–17.2%) and the Middle East (9.8%, 95% CI: 7.8–12.2%). However, this was comparable to the rates observed in the U.S. (15.6%, 95% CI: 12.2–19.7%) and Europe (14.9%, 95% CI: 6.9–29.3%). For advanced neoplasia, Turkey’s prevalence in the younger population (3.2%) also exceeded the rates in East Asia (1.5%) and the Middle East (1.2%), while it aligned with the U.S. (4.0%), and Europe (3.2%) [1,15,16,17,18]. These findings suggest that unique regional risk factors, including dietary patterns, genetic predispositions, and disparities in the healthcare infrastructure, may contribute to the higher prevalence observed in Turkey. In the older population, Turkey demonstrated an EAO-CRN prevalence of 22.9% and advanced neoplasia rate of 7.3%. These rates underscore the importance of maintaining robust screening programs for individuals aged 50–54 years while addressing the growing burden of CRC in younger cohorts. Tailored interventions and screening policies that account for regional disparities and population-specific risks are critical in optimizing CRC prevention strategies.

This research focused on the 45–49 and 50–54 age groups because worldwide patterns show an increasing incidence of early-onset colorectal cancer (EOCRC) in people younger than 50 years [5,6]. Recent studies have reported that EOCRC incidence is disproportionately increasing in these age groups, with a substantial proportion of cases presenting at an advanced stage [5,6]. Similarly, Li et al. and Ong et al. highlighted that countries implementing earlier screening strategies have observed improved early detection rates and patient outcomes [7,8]. These findings support the need for targeted screening in younger populations, especially in regions with an increasing incidence of EOCRC. The increasing incidence of CRC in younger populations underscores the need to tailor screening strategies based on regional and demographic characteristics. Studies have demonstrated that implementing screening at age 45, particularly for high-risk groups, can improve cost-effectiveness and prevent the progression to invasive CRC [9,10]. While our findings suggest that routine screening may not be necessary for all individuals aged 45–49 years, decisions should consider individual risk factors, such as family history, lifestyle, and comorbidities.

The cost-effectiveness of lowering the colorectal cancer screening age to 45 years has been a subject of debate, with studies showing varying economic outcomes depending on the healthcare infrastructure, screening adherence, and CRC incidence rates. High-income countries have demonstrated favorable cost–utility ratios for initiating screening at 45 years due to reduced late-stage treatment costs and improved quality-adjusted life-years (QALYs) [11,12]. However, the economic implications for middle-income countries, including Turkey, remain uncertain and require further investigation. Studies from Thailand and Ukraine suggest that earlier screening can be cost-effective in middle-income settings, but implementation depends on healthcare infrastructure and screening adherence rates [11,13]. In Turkey, preliminary findings indicate that earlier CRC screening improves early detection while reducing long-term treatment expenditures [19,20]. Given the rising incidence of EOCRC and the economic burden of advanced-stage CRC treatment, early screening could potentially reduce long-term healthcare expenditure [5,14]. However, formal cost-effectiveness analyses incorporating direct medical costs, budget impact, and QALY assessments are needed to evaluate the financial sustainability of adjusting national screening policies. Our findings highlight the need for targeted CRC screening policies in middle-income countries, particularly in regions with rising EOCRC incidence. Adapting cost-effective screening models from high-income settings must consider regional differences in healthcare accessibility, population risk factors, and screening adherence rates.

Our results demonstrated that smaller polyps (<5 mm) were significantly more frequently detected in the 45–49 age group than in the 50–54 age group. By contrast, larger polyps (≥10 mm) were more prevalent in the older cohort than in the younger cohort. These findings align with those of previous studies indicating that early-onset colorectal neoplasia is more likely to present with smaller lesions at initial detection, supporting the rationale for early screening in younger populations [15,16,17]. The anatomical distribution of polyps differed significantly across age groups; right-sided (proximal) colon involvement was more common in the 50–54 years age group than in the younger cohort (19.1%). Conversely, rectal localization was significantly higher in the younger group, consistent with global trends showing that EOCRC has a predilection for left-sided and rectal involvement, whereas late-onset CRC tends to develop in the proximal colon [18,21]. This shift may be linked to different molecular pathways, with microsatellite instability more frequently observed in right-sided lesions and chromosomal instability being predominant in left-sided CRC [22,23]. The most common histological subtype in both groups was tubular adenoma, accounting for 84.0% of cases in the 45–49 years age group and 88.4% in the 50–54 years age group. However, tubulovillous adenomas were significantly more frequent in the older cohort, while villous adenomas were exclusively found in the younger age group. Given that villous and tubulovillous adenomas have higher malignant potential, these findings emphasize the need for close surveillance of younger patients presenting with these high-risk histological types [24,25]. These findings suggest that younger individuals present with more distal lesions and smaller polyps, while older patients exhibit a shift towards larger, right-sided lesions with advanced histological features. This has direct implications for screening strategies, where flexible sigmoidoscopy may be particularly beneficial in younger patients, whereas full colonoscopy remains essential for detecting right-sided lesions in older individuals [21,26].

## 5. Limitations and Future Directions

This study has several limitations that should be considered when interpreting the findings. The retrospective design inherently introduces selection bias, limiting our ability to establish causative relationships, and reliance on existing medical records increases the risk of incomplete or missing data, particularly for unmeasured confounders, such as smoking history, dietary patterns, physical activity levels, and other lifestyle factors. These limitations may have influenced the accuracy of the risk stratification and generalizability of our findings.

This study did not include individuals aged 40–44 years, as routine CRC screening is not performed in asymptomatic individuals within this age range in Turkey. This study excluded individuals with a family history of CRC, which may have led to an underestimation of the actual burden of EAO-CRN.

Another limitation was the absence of a comprehensive cost-effectiveness analysis. While preliminary studies have suggested the potential economic benefits of early CRC screening in Turkey, data reliability and international comparability remain uncertain.

To address these limitations, future studies should focus on prospective cohort designs to validate our findings and reduce selection bias. Large-scale longitudinal studies incorporating detailed demographic, genetic, and lifestyle data and cost-effectiveness analyses are essential for refining risk prediction models and improving early screening strategies.

## 6. Conclusions

This study highlights the prevalence of EAO-CRN and EAO-aCRN in individuals aged 45–49 years compared with those aged 50–54 years in a Turkish cohort. The findings revealed that while the detection rates of polyps, adenomas, and advanced polyps were significantly lower in the younger population, the prevalence of early-onset and advanced lesions was still substantial. Specifically, the younger population demonstrated a 16.6% prevalence of EAO-CRN and 3.2% prevalence of advanced neoplasia, exceeding the global average for this age group. These results align with global trends, indicating an increasing incidence of CRC in younger individuals, supporting ongoing discussions on lowering the screening age to 45 years.

## Figures and Tables

**Table 1:** Demographic data of asymptomatic patients.

		Group 1	Group 2	
		n (%)	n (%)	*p* †
Gender	Male	530 (61.5)	1882 (61.1)	0.839
	Female	332 (38.5)	1198 (38.9)	
Polyp Localization	Right colon	27 (19.1%)	168 (23.9%)	<0.001 ***
	Left colon	67 (47.5%)	188 (26.7%)	
	Rectum	23 (16.3%)	128 (18.2%)	
	Multiple	24 (17.0%)	220 (31.3%)	
Dysplasia degree	Low-grade	73 (83.9)	314 (80.3)	0.406
	High-grade	8 (9.2)	31 (7.9)	
	Malign	6 (6.9)	46 (11.8)	
Adenomatous histology	Tubular	68 (84.0)	305 (88.4)	0.012 *
	Tubulovillous	11 (13.6)	40 (11.6)	
	Villous	2 (2.5)	0 (0.0)	
Non-adenomatous histology	Inflammatory	10 (20.0)	72 (26.0)	0.094
	Hyperplastic	37 (74.0)	201 (72.6)	
	Leiomyoma	3 (6.0)	4 (1.4)	
Serrated polyps	Serrated	6 (4.2)	36 (5.1)	0.645
	Non-serrated	137 (95.8)	668 (94.9)	
Polyp size	<5 mm	57 (48.9%)	239 (34.0%)	0.02 *
	5–9 mm	37 (31.8%)	248 (35.2%)	
	≥10 mm	23 (19.3%)	217 (30.7%)	
		Group 1	Group 2	
		Mean ± sd	Mean ± sd	*p* ‡
BMI		28.97 ± 3.99	29.15 ± 3.95	0.200
Polyp size		6.5 ± 5.1	8.8 ± 8.4	<0.001 ***
Polyp number		1.5 ± 0.8	1.9 ± 1.6	0.023 *

BMI: Body Mass Index, SD: standard deviation, †: chi-square test, ‡: Mann–Whitney U test, * *p* < 0.05, *** *p* < 0.001

**Table 2:** Polyp, adenoma, and advanced polyp detection rates between groups.

	Polyp DetR	*p* ‡	Adenoma DetR	*p* ‡	Advanced Polyp DetR	*p* ‡
Group 1	16.6% 22.9%	<0.001 ***	10.8% 13.9%	0.018 **	3.2% 7.3%	<0.001 ***
Group 2						

DetR: detection rate; ‡: chi-square test; ** *p* < 0.01, *** *p* < 0.001.

**Table 3:** Analyses of polyp development and histological subtypes in individuals aged 45–49 years.

Variable	OR	95% CI	*p* Value
Polyp development	0.515	0.434–0.611	<0.001
Adenomatous polyp development	0.643	0.513–0.806	<0.001
Hyperplastic polyps	0.871	0.575–1.321	0.517
Inflammatory polyps	0.922	0.622–2.375	0.685
Tubular adenoma	1.154	0.849–1.568	0.361
Tubulovillous adenoma	1.268	0.677–2.375	0.458
Serrated adenoma	0.490	0.210–1.145	0.099
Low-grade dysplasia	1.028	0.745–1.418	0.869
High-grade dysplasia	1.525	0.779–2.983	0.218
Malignant lesions	0.836	0.438–1.598	0.589
Left colon polyp development	1.699	1.221–2.363	0.020
Right colon polyp development	1.075	0.726–1.592	0.718
Rectal polyp development	0.715	0.489–1.045	0.083
Multiple-site polyp development	0.702	0.497–0.991	0.044
Polyp size 0–4 mm	1.374	0.988–1.910	0.059
Polyp size 5–9 mm	1.331	0.974–1.819	0.073
Polyp size ≥10 mm	0.476	0.330–0.686	<0.001

Multivariate and univariate logistic regression.

## Data Availability

The data of this study are available from the corresponding author upon reasonable request.
